# Identification of Pneumococcal Factors Affecting Pneumococcal Shedding Shows that the *dlt* Locus Promotes Inflammation and Transmission

**DOI:** 10.1128/mBio.01032-19

**Published:** 2019-06-18

**Authors:** M. Ammar Zafar, Alexandria J. Hammond, Shigeto Hamaguchi, Weisheng Wu, Masamitsu Kono, Lili Zhao, Jeffrey N. Weiser

**Affiliations:** a Department of Microbiology and Immunology, Wake Forest School of Medicine, Winston-Salem, North Carolina, USA; b Department of Microbiology, New York University, New York, New York, USA; c Division of Infection Control and Prevention, Osaka University Hospital, Osaka, Japan; d BRCF Bioinformatics Core, University of Michigan, Ann Arbor, Michigan, USA; e Department of Otolaryngology-Head and Neck Surgery, Wakayama Medical University, Wakayama, Japan; f Department of Biostatistics, University of Michigan, Ann Arbor, Michigan, USA; Carnegie Mellon University

**Keywords:** bacterial transmission, *Streptococcus pneumoniae*, host-pathogen interactions, inflammation, transposon mutagenesis

## Abstract

Host-to-host transmission is a necessary but poorly understood aspect of microbial pathogenesis. Herein, we screened a genomic library of mutants of the leading respiratory pathogen Streptococcus pneumoniae generated by mariner transposon mutagenesis (Tn-Seq) to identify genes contributing to its exit or shedding from the upper respiratory tract (URT), the limiting step in the organism’s transmission in an infant mouse model. Our analysis focused on genes affecting the bacterial surface that directly impact interactions with the host. Among the multiple factors identified was the *dlt* locus, which adds d-alanine onto lipoteichoic acids (LTA) and thereby increases Toll-like receptor 2-mediated inflammation and resistance to antimicrobial peptides. The more robust proinflammatory response in the presence of d-alanylation promotes secretions that facilitate pneumococcal shedding and allows for transmission. Expression of the *dlt* locus is controlled by the CiaRH system, which senses cell wall stress in response to antimicrobial activity, including in response to lysozyme, the most abundant antimicrobial along the URT mucosa. Accordingly, in a *lysM^−/−^* host, there was no longer an effect of the *dlt* locus on pneumococcal shedding. Thus, our findings demonstrate how a pathogen senses the URT milieu and then modifies its surface characteristics to take advantage of the host response for transit to another host.

## Abstract (precis)

Streptococcus pneumoniae (the pneumococcus) is a common cause of respiratory tract and invasive infection. The overall effectiveness of immunization with the organism’s capsular polysaccharide depends on its ability to block colonization of the upper respiratory tract and thereby prevent host-to-host transmission. Because of the limited coverage of current pneumococcal vaccines, we carried out an unbiased *in vivo* transposon mutagenesis screen to identify pneumococcal factors other than its capsular polysaccharide that affect transmission. One such candidate was expressed by the *dlt* locus, previously shown to add d-alanine onto the pneumococcal lipoteichoic acid present on the bacterial cell surface. This modification protects against host antimicrobials and augments host inflammatory responses. The latter increases secretions and bacterial shedding from the upper respiratory tract to allow for transmission. Thus, this study provides insight into a mechanism employed by the pneumococcus to successfully transit from one host to another.

## INTRODUCTION

Successful pathogens require the ability to transit to new hosts. Studying host-to-host transmission within an experimental setting, however, has proven to be problematic because of a lack of tractable, representative animal models and the inherent complexities of the steps involved (exit from the host, transit and survival in the environment, and establishment in a new host). As a result, we have limited biological insight into the contributions of either bacterial or host factors in a pathogen’s dissemination.

Our current understanding of host-to-host transit comes mainly from epidemiological studies that address modes of transmission (1–6). The transmission route utilized by one of the leading bacterial pathogens, Streptococcus pneumoniae (the pneumococcus), is via the nasal secretions of colonized individuals and requires close contact (7, 8). Although colonization of the nasopharynx is generally considered asymptomatic (the carrier state), under certain circumstances pneumococci can invade sterile sites within the host, resulting in disease manifestations, including otitis media, pneumonia, sepsis, and meningitis (8, 9). Colonization and transmission are most common among young children, especially in crowded settings, such as day care centers, or in association with episodes of viral upper respiratory tract infection (8, 10, 11).

Each year, approximately a million individuals succumb to infections associated with S. pneumoniae (12, 13). For this reason, the World Health Organization has labeled S. pneumoniae a priority pathogen, a designation that emphasizes the need for new strategies to combat its spread (14). Due to the challenges of studying pneumococcal transmission, most studies have focused on its disease manifestations (within-host events), where significant advances have broadened our understanding of pneumococcal pathogenesis (7). Recently, we utilized an infant mouse model to study the biology of pneumococcal transmission (the between-host events) (15). Collectively, these studies demonstrate that infection induces a mild acute inflammatory response on the mucosal surfaces of the upper respiratory tract (URT) that promotes nasal secretions and increases the numbers of pneumococci shed from colonized pups (16). A high level of bacterial shedding is required to overcome the tight population bottleneck observed during intralitter transit from colonized to uncolonized pups (17).

We recently showed that the two major virulence determinants of the pneumococcus also contribute to its transmission among infant mice. Pneumolysin, the organism’s sole toxin that forms pores in host membranes, facilitates transmission by enhancing mucosal inflammation and the frequency of high-shedding events (18). We also observed that certain pneumococcal capsule types are transmitted at higher rates and that this effect is independent of the genetic background (19). This role of capsule type correlates with its effect on shedding and the ability of the organism to escape from entrapment from negatively charged URT mucus.

The purpose of this study was the high-throughput identification of the complete array of pneumococcal factors that affect its transmission using the infant mouse model. We used the technique of mariner transposon mutagenesis (Tn-Seq) to screen a genomic transposon library to identify loci that negatively affect pneumococcal shedding, the limiting step in its transmission (16, 17, 20). Many of the genes identified impact the bacterial surface or host environment. In this report, we focused on one such factor, the *dlt* locus, which modifies teichoic acids through d-alanylation (21, 22). We show that the *dlt* locus promotes shedding and transmission by affecting URT inflammation by altering signaling in a Toll-like receptor 2 (TLR2)-dependent manner. Moreover, this effect of Dlt-mediated d-alanylation correlated with decreased sensitivity to the abundant URT antimicrobial lysozyme.

## RESULTS

### Identification of pneumococcal genes that affect transmission of the pneumococcus.

**FIG 1:**
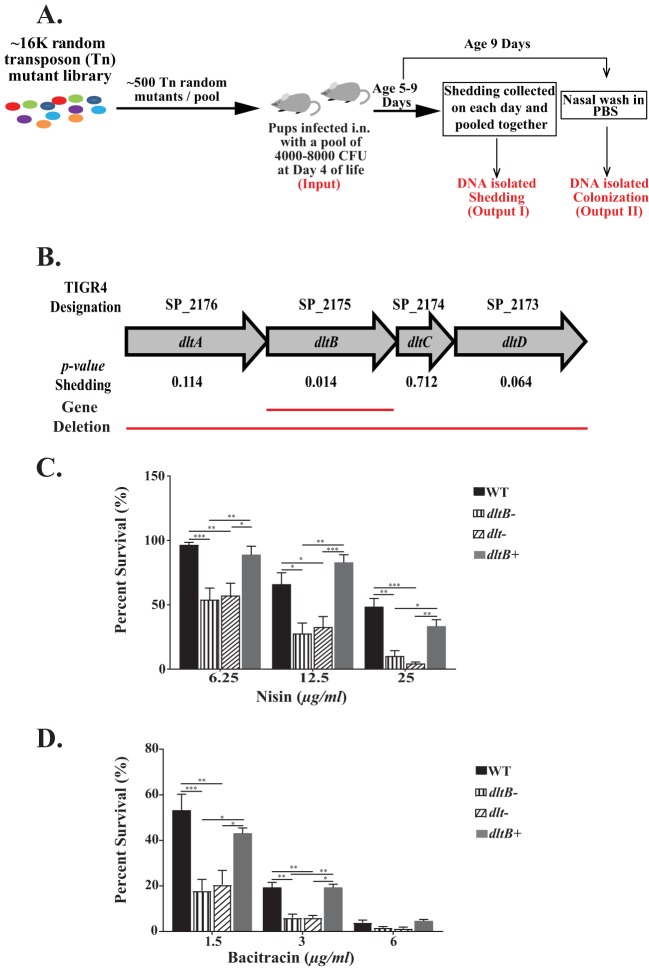
(A) Schematic representation of the mariner transposon mutagenesis screen (Tn-Seq) carried out *in vivo* in infant mice (pups). Pups at day 4 of life were infected with 4,000 to 8,000 CFU of a random mutant pool (∼500 mutants), and the bacteria shed daily from nasal secretions were collected, quantified, and pooled from days 5 to 9. DNA was isolated from the pooled samples (output I). At 9 days of age, URT lavage fluids in PBS were obtained to quantify the colonization density and DNA isolated (output II). (B) The genetic organization of the genes within the *dlt* locus of S. pneumoniae with the TIGR4 designation and their names are listed. Adjusted *P* values for shedding for each gene are listed, and the solid red lines represent the gene deletions constructed for the current study. (C and D) Survival assay using S. pneumoniae strain TIGR4 Strep^r^ (T4S), the *dltB*- and *dlt*-negative mutants, and the corrected strain (the *dltB^+^* strain) in the presence of various concentrations of nisin or bacitracin. Survival was quantified by incubating serially diluted bacteria in tryptic soy broth with the indicated concentration of the antimicrobial peptide for 3 h at 37°C in 5% CO_2_. Each strain was independently tested at least three times, with statistically significant differences being calculated using the Kruskal-Wallis analysis of variance with Dunn’s posttest. *, *P* ≤ 0.05; **, *P* ≤ 0.01; ***, *P* ≤ 0.001.

To identify genes that potentially impact pneumococcal transmission, we used mariner transposon mutagenesis (Tn-Seq) to generate a library of ∼16,000 random mutants in a streptomycin-resistant derivative of strain TIGR4 (T4S) (Fig. 1A). In total, 28 pools, each containing ∼500 random mutants, were screened in infant mice (pups). Each pool (4,000 to 8,000 CFU) was inoculated intranasally (i.n.) into two 4-day-old infant mice. Samples obtained from each of the pups were kept separate and considered biological replicates for each pool. Shedding was used as a proxy for transmission because of the tight population bottleneck between hosts and its close correlation with the rate of transmission (15, 17–19). Shedding was quantified daily over 5 days (days 5 to 9), colonies were pooled, and DNA was isolated (output I). At day 9 of life, nasal lavage fluid specimens were collected from the URT and placed in phosphate-buffered saline (PBS) to determine the colonization density and DNA isolated (output II). Isolated DNA from the outputs was used as a template for PCR amplification and addition of unique sequence tags to distinguish the different outputs. These samples were processed as described in detail in the Materials and Methods section. Input pools were processed in a similar manner.

Data analysis was carried out by using TnseqDiff, which utilizes a two-step approach in determining the conditional essentiality of a gene (the output compared to the input) (23). To identify factors that affect shedding, genes revealed through the TnseqDiff approach were further filtered to find candidates defective in shedding that did not impact colonization by using an arbitrary cutoff of an adjusted *P* value of <0.05 for shedding and an adjusted *P* value of >0.05 for colonization. Using these parameters, there were >300 candidates that negatively affected pneumococcal shedding (see Table S1 in the supplemental material). No pneumococcal genes that positively impacted shedding were identified. There were an average of >10 unique transposon insertions per open reading frame.

10.1128/mBio.01032-19.1 TABLE S1 List of Streptococcus pneumoniae genes involved in shedding. Genes that had an adjusted *P* value of >0.05 for colonization and an adjusted *P* value of <0.05 for shedding were considered candidates for a role in pneumococcal shedding. Download Table S1, XLSX file, 0.04 MB. Copyright © 2019 Zafar et al. 2019 Zafar et al. This content is distributed under the terms of the Creative Commons Attribution 4.0 International license.

As the pneumococcal surface is dynamic and interfaces with the host, further analyses focused on candidates that modify its surface or the surrounding host environment. This targeted approach revealed a list of 20 candidate genes (Table 1) whose functions included (i) the biosynthesis of capsular polysaccharide, which was previously implicated in shedding (19), (ii) adherence (pilus attachment, choline binding proteins), and (iii) interactions with glycans in host glycoconjugates and mucus (deglycosylation and mucin binding).

**TABLE 1:** Surface-exposed or surface-acting factors in pneumococcal shedding^*a*^

TIGR4 designation	*P* value for shedding	Gene name and description	Function (reference)
SP_0112	0.019949	*artP*; amino acid ABC transporter	Arginine ABC transporter (64)
SP_0148	0.000324	*gshT*; ABC transporter	Predicted glutathione ABC transporter (65)
SP_0198	0.030898	Hypothetical protein	Lipoprotein, putative d-stereospecific aminopeptidase (44, 45)
SP_0314	0.013614	*hysA*; hyaluronate lyase	Surface enzyme that cleaves hyaluronan, a component of the host extracellular matrix (66, 67)
SP_0352	0.000993	*cps4G*; glycosyltransferase	Biosynthesis of capsular polysaccharide (68)
SP_0368	0.000004	*eng*; endo-alpha-*N*-acetylgalactosaminidase	Secreted O-glycosidase that modifies host O-linked glycans (69)
SP_0391	0.001690	*cbpF*; choline binding protein F	Modulates the autolytic function of LytC (70)
SP_0467	0.036190	*srtC*; sortase C	Involved in covalent attachment of the pilus to the peptidoglycan cell wall (71, 72)
SP_0614	0.016540	*estA*; esterase	Enhances neuraminidase activity by removing acetylation from sialic acid (73)
SP_0667	0.000046	*cbpL*; choline binding protein L	Contributes to resistance against phagocytosis and is involved in adhesion (74)
SP_0771	0.021157	*slrA* (*ppiA*); peptidyl-prolyl *cis*-*trans*-isomerase, cyclophilin type	Catalyzes isomerization of proline containing tetrapeptides (75)
SP_0899	0.037124	Conserved hypothetical protein	Surface-exposed lipoprotein of unknown function (76)
SP_0965	0.023346	*lytB*; endo-beta-*N*-acetylglucosaminidase	Nonautolytic peptidoglycan hydrolase, acts as a glucosaminidase and is involved in cell division cycle (77)
SP_1000	0.016753	*etrx2*; thioredoxin family protein	Surface-exposed lipoprotein that provides oxidative stress resistance (78)
SP_1004	0.000050	*phtE*; histidine triad protein	Surface-exposed protein that promotes adherence to host cell surfaces (79)
SP_1154	0.014494	*zmpA*; zinc metalloproteinase	Cleaves human immunoglobulin A (IgA1) (80, 81)
SP_1492	0.035625	*mucBP*; cell wall surface anchor family protein	Binds mucin, has a potential role in pneumococcal adherence (82, 83)
SP_1573	0.000314	*lytC*; lysozyme	Surface-bound lysozyme that acts as a pneumococcal cell wall hydrolase (84)
SP_1796	0.045895	*fusA* (*susX*); carbohydrate ABC transporter, substrate binding protein	Required for utilization of fructose oligosaccharide, uses inulin as a carbon source (85–87)
SP_1872	0.034338	*piuA*; iron compound ABC transporter, iron compound-binding protein	Lipoprotein component of iron ABC transport Piu system (88, 89)
SP_1963	0.036813	Putative hemolysin	CBS domain-containing protein with homology to putative cytolysin proteins (64)
SP_1964	0.033659	*endA*; DNA entry nuclease	Surface endonuclease that degrades the DNA scaffold of neutrophil extracellular traps (NETs) (90)
SP_2084	0.041972	*pstS*; phosphate ABC transporter, phosphate-binding protein	Subunit of the phosphate ABC transporter, implicated in penicillin resistance (91, 92)
SP_2099	0.003095	*pbpIB*; bifunctional penicillin-binding protein	Synthesis of peptidoglycan (transglycosylation and transpeptidation) (93)
SP_2175	0.014521	*dltB*; membrane protein involved in d-alanine export	Addition of d-alanine to teichoic acids (27)

a Streptococcus pneumoniae genes identified by mariner transposon mutagenesis (Tn-Seq) screening to be involved in pneumococcal shedding and predicted to be either surface-exposed or surface-acting factors.

### Identification and analysis of the *dlt* locus.

A candidate of particular interest was the gene *dltB* (Table 1), which forms part of the *dlt* locus (*dltA*, *dltB*, *dltC*, and *dltD* [*dltA-D*]). The Dlt pathway modifies the anionic glycopolymer teichoic acid (TA), a prominent surface feature of Gram-positive bacteria, by the addition of d-alanine onto its ribitol or glycerol (in Staphylococcus aureus) backbone (21, 22, 24). In Bacillus subtilis, inactivation of the Dlt pathway prevents d-alanylation of both lipoteichoic acid (LTA) and wall teichoic acid (WTA) (25). d-Alanylation has the effect of decreasing the negative charge of TAs, which could alter interactions with host factors and inflammatory responses. We were intrigued by the potential role of the Dlt pathway in altering the host inflammatory response, as our previous report demonstrated that expression of the pneumococcal cytolysin, pneumolysin, causes an inflammatory response that enhances shedding and transmission (18).

DltB, identified in our shedding screen, is proposed to function as a chaperone in the secretion of the d-alanine carrier protein (DltC) (26). To confirm that the *dlt* locus impacts pneumococcal shedding, we constructed a mutant with an unmarked, in-frame deletion of *dltB* (the *dltB*-negative [Δ*dltB*] mutant) and also a mutant with complete in-frame knockout of the entire *dlt* locus (the *dltA-D*-negative [Δ*dltA-D*] mutant) (Fig. 1B).

Previous studies showed that d-alanylation of the TA of S. pneumoniae results in increased resistance to cationic antimicrobial peptides (AMP), in an effect thought to be related to the altered surface charge of d-alanylation (27). To validate the phenotype of our constructs, we assessed resistance to the cationic AMP nisin, which has previously been shown to be affected by *dlt* (27), and confirmed that interruption of the pneumococcal *dlt* locus decreased survival in the presence of nisin (Fig. 1C). The *dlt* mutants were also more sensitive to the cyclic antimicrobial peptide bacitracin. This suggests a more general effect of Dlt-mediated cell surface modification, since bacitracin acts by a different mechanism: the disruption of peptidoglycan biosynthesis (28) (Fig. 1D). In the presence of both of these antimicrobials, the survival defect was specific to the *dlt* locus, as correction of the genotype (producing the *dltB*-positive [*dltB*^+^] mutant) restored the wild-type (WT) phenotype.

### Contribution of the *dlt* locus to pneumococcal shedding and transmission.

**FIG 2:**
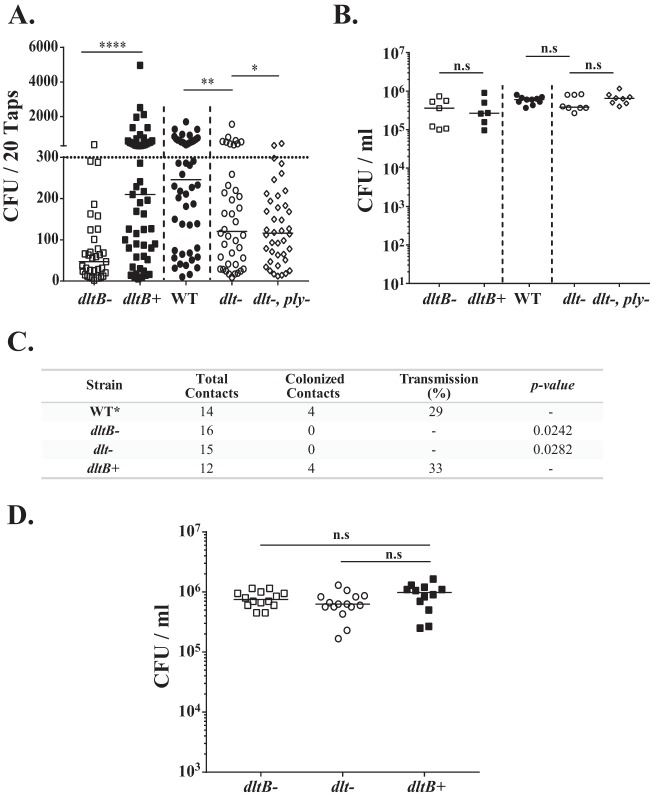
The *dlt* locus affects pneumococcal shedding and transmission without impacting colonization. (A) Pups were challenged i.n. at 4 days of age with the indicated construct, and bacteria shed daily from nasal secretions were collected and quantified from days 5 to 9. Median values are indicated, and each symbol represents the number of CFU measured from a single pup. The dotted horizontal line represents the 300-CFU threshold described in Results. The dashed vertical line separates the intralitter comparisons of two pneumococcal strains. Data are for 6 to 10 pups per group. (B) Colonization density of each pneumococcal construct in cultures of URT lavage fluids obtained from pups at 9 days of age. Median values are shown. (C) Summary of the rate of transmission of *dlt* locus mutants and the corrected strain (the *dltB^+^* mutant) from colonized index pups at the age of 4 days to naive contact pups in the same litter. The transmission rate was determined by the number of contact pups (at a 1:1 ratio to index mice) colonized by S. pneumoniae at the age of 14 days. Data for the WT are historic data (15). *dlt* locus mutants were compared to the *dltB*^+^ group by using Fisher's exact test. (D) Colonization density of each pneumococcal *dlt* construct tested for transmission (index mice) in cultures of URT lavage fluids obtained from pups at 14 day of age. *, *P ≤ *0.05; **, *P* ≤ 0.01; ****, *P ≤ *0.0001, n.s, not significant.

Because of day-to-day variation, shedding was measured daily (days 1 to 5 postinoculation) and values were pooled for comparison (15). Both the *dltB*- and *dlt*-negative mutants showed reduced median shedding compared to either the corrected mutant (the *dltB*^+^ mutant) or the WT type 4 strain (Fig. 2A). These results included intralitter comparisons of strains from pups from multiple litters to control for host and environmental effects on shedding. The contribution of the *dlt* locus to shedding was not due to differences in colonization density, as all the strains colonized equally well at the conclusion of the shedding experiment at age 9 days (Fig. 2B).

Next, we determined whether the attenuated shedding seen in *dlt* mutants leads to reduced transmission. Using a 1:1 ratio of pups colonized by inoculation (index pups) to uninoculated littermates (contact pups), no transmission events were detected with either the *dltB*- or *dlt*-negative strains that showed diminished shedding (Fig. 2C). Importantly, transmission to levels previously reported for the WT strain was restored by correction of the deletion in *dltB* (producing the *dltB*^+^ strain) (15, 18). Again, all constructs colonized pups at a high density at the conclusion of the transmission experiment at age 14 days (Fig. 2D). Thus, we validated the initial observations from the Tn-Seq screen and confirmed that the *dlt* locus does not impact colonization but is needed for sufficient pneumococcal shedding to allow for host-to-host transmission.

Because expression of the pneumolysin toxin (Ply) also increases shedding and transmission (18), we examined whether the *dlt* locus had an additive effect with Ply. The *dlt*- and *ply*-negative double mutant showed low median shedding that was indistinguishable from that of the *dlt*-negative single mutant (Fig. 2A). The major effect of the loss of *ply*, however, was fewer high-shedding events (>300 CFU) rather than altered median shedding (18). The proportion of high-shedding events was significantly lower for the *dlt*- and *ply*-negative double mutant than for the *dlt*-negative single mutant, suggesting that the toxin and d-alanylation may act through different pathways to induce shedding.

### Role of d-alanylation in URT inflammation.

**FIG 3:**
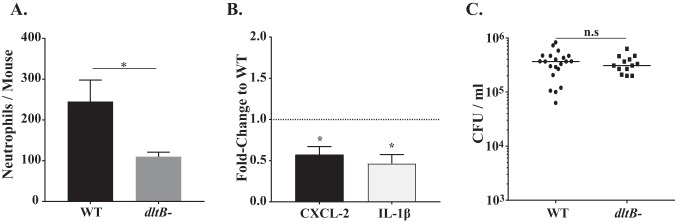
The absence of d-alanylation (*dltB* negative) leads to reduced URT inflammation. (A) Number of neutrophils determined by flow cytometry (CD45^+^, CD11b^+^, and Ly6G^+^ events) in URT lavage fluids obtained from WT (T4S)-inoculated or isogenic *dltB*-negative construct-inoculated pups at age 7 days. Values are ±SEM (*n* = 8 to 11). (B) Gene expression in pups colonized with the *dltB*-negative mutant relative to that in WT strain-inoculated pups at age 7 days, as measured by qRT-PCR, for the chemokine/cytokine shown. Values are ±SEM (*n* > 10). (C) Colonization density in URT lavage fluids obtained at age 7 days, with the median values being shown. *, *P* ≤ 0.05; n.s, not significant.

*In vitro* studies have shown that LTA, once it is released after bacteriolysis, triggers an inflammatory response via recognition by the pathogen recognition receptor Toll-like-receptor 2 (TLR2) (29–31) and that d-alanylation of pneumococcal LTA augments TLR2-mediated inflammation (31, 32). Therefore, we postulated that disruption of the *dlt* locus might impact shedding by dampening the proinflammatory responses of the URT during colonization. As predicted, at age 7 days (2 days postchallenge), the numbers of neutrophils (CD45^+^, Ly6G^+^, and CD11b^+^ events) detected by flow cytometry of the nasal lavage fluid specimens from infant mice infected with the *dltB*-negative mutant were reduced compared to the numbers for infant mice infected with the WT strain (Fig. 3A). Additionally, there was decreased URT transcription of the chemokine CXCL-2, which stimulates neutrophil recruitment, and the proinflammatory cytokine interleukin-1β (IL-1β) in the lavage fluid specimens obtained from infant mice colonized with the *dltB*-negative mutant (Fig. 3B). The observed differences in the inflammatory response were not due to differences in colonization density, as both strains colonized to a high density at this earlier time point (Fig. 3C). Thus, our *in vivo* data correlated with the findings of the *in vitro* studies linking d-alanylation to increased inflammation and provided further evidence correlating inflammation and shedding.

### The effect of d-alanylation of LTA on shedding requires signaling through TLR2.

Next, we examined if the increased inflammation observed with strains expressing d-alanylated TAs was due to increased TLR2-mediated signaling, as suggested by the *in vitro* studies.

**FIG 4:**
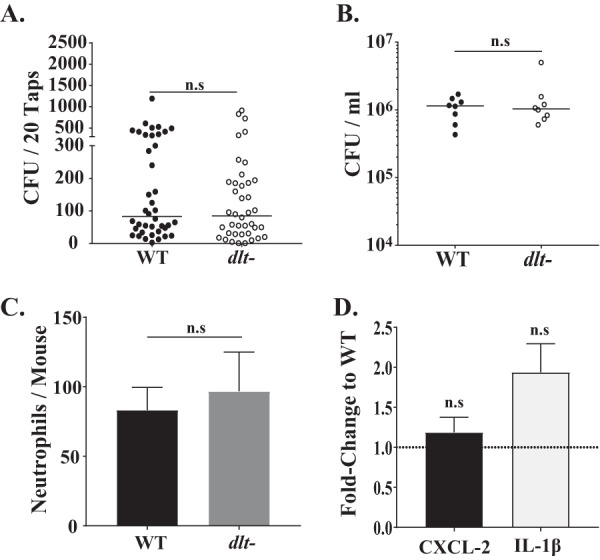
The pneumococcal *dlt* locus is epistatic to the host pattern recognition receptor TLR2. (A) Congenic *tlr2^−/−^* pups were colonized at age 4 days with the WT strain (T4S) or with its isogenic *dlt*-negative construct. Daily shedding in nasal secretions was quantified from ages 5 to 9 days, with the median values being indicated and with each symbol representing the number of CFU observed from a single pup on a single day. Data are for 8 pups per group. (B) Colonization density in URT lavage fluids obtained at age 9 days, with the median value being shown. (C) Number of neutrophils determined by flow cytometry (CD45^+^, CD11b^+^, and Ly6G^+^ events) in URT lavage fluids obtained from *tlr2^−/−^* pups inoculated with either the WT strain (T4S) or its isogenic *dlt*-negative construct at age 7 days. Values are ±SEM (*n* = 8 to 11 pups/group). (D) Gene expression in colonized *tlr2^−/−^* pups infected with the *dlt*-negative construct relative to those infected with the WT (T4S) at age 7 days, as measured by qRT-PCR for the chemokine/cytokine shown. Values are ±SEM (*n* > 8). n.s, not significant.

We previously reported that *tlr2^−/−^* pups show decreased URT inflammation and reduced shedding (18). The difference in median shedding between mice infected with the *dlt*-negative mutant and mice infected with its parent strain, as noted in WT mice, was no longer observed in *tlr2^−/−^* infants, and both strains colonized the knockout mice at equivalent densities (Fig. 4A and B). Moreover, in *tlr2^−/−^* mice infected with the *dlt*-negative mutant or its parent strain, there was no difference in neutrophil numbers or the transcript levels of chemokines/cytokines upregulated by the presence of *dlt* (Fig. 4C and D). Together, our *in vivo* data suggest that d-alanylation of LTA increases inflammation in a TLR2-depentent manner and that this increased host response drives pneumococcal shedding.

### Lysozyme treatment leads to upregulation of the *dlt* locus.

**FIG 5:**
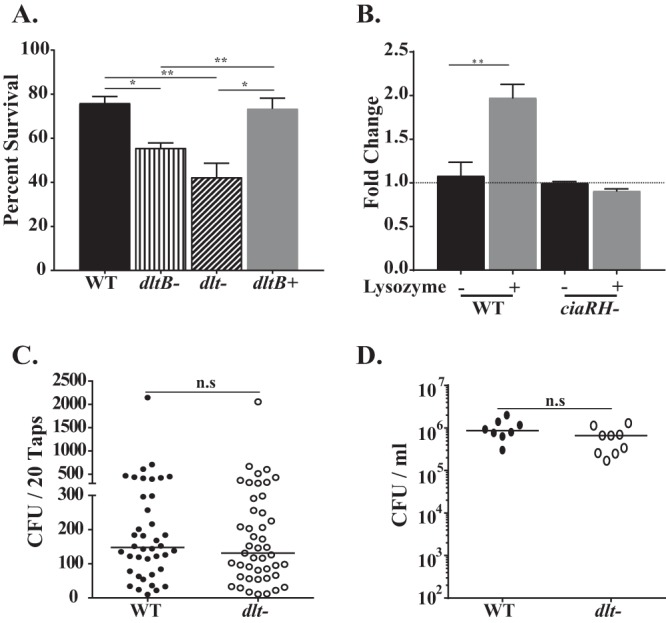
d-Alanylation of pneumococcal lipoteichoic acids provides protection against host lysozyme. (A) Lysozyme survival assay using S. pneumoniae strain TIGR4 (T4S), the *dltB*- and *dlt*-negative mutants, and the corrected strain (the *dltB^+^* strain). Survival was quantified by incubating serially diluted bacteria in tryptic soy broth with 1.0 mg/ml of chicken egg lysozyme for 3 h at 37°C in 5% CO_2_. Each strain was independently tested at least three times. (B) Gene expression of *dltA* for pneumococcal strains treated with or without lysozyme for 60 min in tryptic soy broth at 37°C, as measured by qRT-PCR. (C) Congenic *lysM^−/−^* pups were colonized at age 4 days with the WT strain (T4S) or with its isogenic *dlt*-negative construct using intralitter comparisons. Daily shedding in nasal secretions was quantified from ages 5 to 9 days, with median values being indicated and with each symbol representing the number of CFU observed from a single pup on a single day. Data are for 4 to 5 pups per group. (D) Colonization density in URT lavage fluids obtained at age 9 days, with the median value being shown. *, *P* ≤ 0.05; **, *P* ≤ 0.01; n.s, not significant.

Lysozyme, an antimicrobial enzyme that functions by degrading the bacterial peptidoglycan backbone, is one of the most abundant antimicrobial proteins present on the mucosal surface of the URT (33). To evade lysis by the high concentration of lysozyme in its URT niche, S. pneumoniae, like other mucosal pathogens, must modify its cell wall (34). The *dlt* locus has been reported to confer resistance against lysozyme in diverse bacterial species, such as Bacillus subtilis, Clostridium difficile, and Enterococcus faecalis (26, 35–37). As the Dlt pathway modifies TA, we hypothesized that it might also contribute to the resistance of S. pneumoniae against lysozyme-mediated killing, as observed for bacitracin, another antimicrobial that targets peptidoglycan (Fig. 1D). In the presence of physiologic concentrations of lysozyme in the URT (33), the WT strain was resistant to its lytic effects, but survival was reduced when the *dlt* locus was disrupted (Fig. 5A). Restoration of the intact *dlt* locus (producing the *dltB*^+^ strain) led to WT levels of survival.

It has been suggested that in response to AMPs the CiaRH two-component regulatory system, present in different streptococcal species, upregulates expression of the *dlt* locus (38). Therefore, we examined whether the cell wall stress caused by lysozyme would also lead to Dlt pathway upregulation. As shown in Fig. 5B, we observed a 2-fold induction of the *dlt* locus, as evidenced by an increase in *dltA* transcript levels, that was dependent on CiaRH, since for a *ciaRH*-negative mutant treated with lysozyme, there was no upregulation of *dltA* expression. Our finding suggests that CiaRH-mediated sensing of cell wall perturbation by lysozyme upregulates the Dlt pathway. We then determined whether the contribution of the *dlt* locus required lysozyme by the use of *lysM^−/−^* mice, which lack the enzyme in URT secretions. In the absence of the cell wall stress from lysozyme, when our study predicts that expression of the *dlt* locus would not be upregulated, shedding was lower than that in WT mice and there was no longer an effect of the *dlt* locus (Fig. 5C). Deletion of the *dlt* locus or the lack of lysozyme had no effect on the colonization density (Fig. 5D).

## DISCUSSION

The long-term goal of this project is to provide a thorough understanding of the biology of pneumococcal transmission. Widespread immunization of children with the pneumococcal conjugate vaccine (PCV) since 2000 has led to a dramatic reduction in invasive pneumococcal disease, including for unimmunized groups, such as adults and the elderly (39, 40). This indirect protection, or herd immunity, which accounts for most of the overall efficacy of PCV, has been attributed to reduced carriage and transmission within the community (41, 42). PCV, however, is based on capsular polysaccharide and targets only a limited number of the >95 known serotypes (43). A more comprehensive understanding of pneumococcal surface factors contributing to its transmission, therefore, could yield novel targets that could broaden and make more effective prevention strategies through immunization.

In the current study, we used a genomic approach to identify pneumococcal genes affecting the exit of the pneumococcus from the colonized host (i.e., shedding) through nasal secretions using an infant mouse model. The function and the role of a large proportion of pneumococcal genes in transmission remain to be determined. Shedding was used as a proxy for transmission because of the tight population bottleneck during transmission (17). It was necessary to focus on shedding, an earlier event that is the limiting step in host-to-host transit (15, 17–19). We validated this approach by confirming that pneumococci carrying one of the hits from the Tn-Seq screen, the *dlt* locus, that showed reduced shedding were also attenuated in transmission among infant mice. Besides confirming the effects of the *dlt* locus, to date we have validated the effects of two other hits listed in Table 1, SP_1963 (annotated as a putative hemolysin) and SP_1964 (endonuclease; *endA*). Pneumococci carrying both were confirmed to have a defect in shedding, but they did not impact colonization density. Prior studies using *in vitro* or *in vivo* or screens of random pneumococcal transposon mutants have been used to identify essential genes or within-host factors (virulence factors), whereas our *in vivo* screen is the first to examine factors involved in between-host events (44–47).

There are several important caveats to the approach used in our genomic screen. First, we identified over 300 genes that impact pneumococcal shedding, suggesting that ∼15% of all nonessential TIGR4 genes significantly affect its shedding (output I) and not its colonization (output II). One reason that our screen identified such a high number of genes affecting shedding could be the less stringent cutoff (*P* < 0.05) used because shedding is an inherently variable readout. Additionally, our screen was carried out with a single highly annotated pneumococcal strain (TIGR4), although pneumococcal isolates show marked differences in genome content and transmissibility (19, 48–50). Moreover, mice are not a natural host for the pneumococcus, even though the infant mouse model recapitulates many of the key features of human contagion (need for close contact, increased rate during viral coinfection and among infants). It is also likely that random transposon insertions might only partially abrogate the function of a gene or lead to polar effects, and TnseqDiff accounts only for inserts that occur within coding regions. Finally, *trans*-acting factors, such as the effect of *ply* on inflammation, could be obscured when screening large pools of mutants. This could explain why the effect of the loss of *dlt* appeared to be greater for the constructs (the *dltB*- and *dlt*-negative constructs) tested individually than for the transposon mutant pools.

Taking into account the dynamic nature of the pneumococcal surface and its interactions with the host, we focused on factors that attenuate shedding, thereby reducing transmission, by modifying its surface. This led us to further characterize the *dlt* locus, since pneumococcal LTA triggers a strong TLR2-dependent response that is partially dependent upon its d-alanylation via the Dlt pathway (29–32). Our findings confirmed that mutants disrupting the Dlt pathway showed reduced shedding, a defect that correlated with reduced levels of acute inflammation, with sensing by TLR2 mediating the inflammatory signal. URT inflammation increases the production of mucins, including the major secretory mucin Muc5ac, and the volume of nasal secretions that drive pneumococcal shedding (16). The mechanism of how d-alanylated LTA contributes to recognition through host pattern recognition receptors remains to be determined. In this regard, there remains controversy as to the contribution of TLR2-mediated sensing of LTA. Work by Kang et al. showed that LTA can bind to the TLR2 extracellular domain but not cause heterodimerization (51). This work, however, used only the soluble domains, and the authors suggest that their findings do not exclude the possibility that LTA may induce the dimerization of full-length TLR2 with TLR1 or TLR6 under membrane-attached conditions. Furthermore, Gisch et al. showed that with lipid-free LTA (through hydrogen peroxide treatment or the use of a mutant defective in lipidation of the prelipoproteins [a Δ*lgt* mutant]), IL-8 production *in vitro* was abolished, suggesting that the lipid moiety is important for signaling (52). This report also suggested that lipopeptide-free LTA still had an immunostimulatory effect in human mononuclear cells, supporting the suggestion that the lipid moiety in LTA may still be important for sensing, possibly via TLR2 recognition, an observation consistent with our findings. On the other hand, in Staphylococcus aureus, d-alanine modification of LTA, which is structurally distinct from that of S. pneumoniae, did not similarly affect inflammatory responses (53). It is also possible that the *dlt* locus affects the release of lipoproteins in response to antimicrobial activity, with subsequent sensing via TLR2.

Our previous study demonstrated the importance of TLR2-mediated sensing and the pneumolysin toxin in inflammation, shedding, and transmission but did not establish a link between these pathways. The *dlt*-dependent effects observed in the current study are epistatic to TLR2 sensing but independent of pneumolysin (Fig. 2A). These results suggest that there is more than a single type of inflammatory response to pneumococcal colonization that affects bacterial shedding and transmission. Furthermore, *ply* was not identified in the screen because transposon mutations in this gene affected both shedding and colonization. Since we previously showed that mutants with a nonpolar *ply* in-frame clean deletion have a shedding defect that does not impact colonization, it is possible that the transposon insertions in *ply* have polar effects. Consistent with our previous findings that link shedding levels with the transmission rate, we observed that reduced shedding in the absence of a functional *dlt* locus results in a complete loss of transmission.

Another effect of Dlt-mediated d-alanylation of LTA observed in our study was increased resistance to the antimicrobial actions of lysozyme. This result adds to the list of pneumococcal enzymes, including *pgdA* and *adr*, that modify the pneumococcus cell surface to evade lysis by this highly abundant host factor found in URT secretions (34, 54–56). Our findings obtained with bacitracin, which hinders peptidoglycan biosynthesis, and lysozyme, which cleaves the peptidoglycan backbone, show that the reduced surface charge by d-alanylation acts in a manner that aids in protecting the pneumococcus against both cationic AMPs and other agents that perturb the integrity of the cell surface. A recent report on group B streptococcus suggests that d-alanylation increases the cell wall density by changing the conformation of the LTA in a manner that can limit penetration by AMPs and, thus, increase resistance and survival (57). Furthermore, during treatment with physiological levels of lysozyme, there is a CiaRH-dependent upregulation in expression of the Dlt pathway, suggesting a programmed response to cell wall stress. It appears that when targeted by antimicrobial peptides/proteins, such as lysozyme, pneumococci increase d-alanylation of LTA, which enhances TLR2-mediated inflammation. This increased URT inflammation in turn facilitates increased shedding to allow for exit of the organism to establish itself in a new, more hospitable host. Thus, our study demonstrates that the pneumococcus is a highly adaptable pathogen capable of modifying its cell surface in response to the host environment to promote both its survival and its dissemination.

## MATERIALS AND METHODS

### Ethics statement.

This study was conducted according to the guidelines outlined by National Science Foundation animal welfare requirements and the *Public Health Service Policy on Humane Care and Use of Laboratory Animals* (58). The New York University Medical Center IACUC oversees the welfare, well-being, and proper care and use of all vertebrate animals.

### Growth conditions and strain construction.

Pneumococcal strains were grown statically in tryptic soy (TS) broth (Becton, Dickinson [BD]) at 37°C. Upon reaching the desired optical density at 620 nm (OD_620_), cells were washed and diluted in sterile phosphate-buffered saline (PBS) for inoculation. For quantitative culture, serial dilutions were plated on TS agar-streptomycin (200 μg/ml) supplemented with either 5% sheep blood or catalase (6,300 U/plate; Worthington Biochemical Corporation) and incubated overnight at 37°C with 5% CO_2_. A streptomycin-resistant derivative of the type 4 strain TIGR4 (T4S), P2406, was used throughout the study (15).

The *dltB* knockout strain was constructed in a two-step process using the Janus cassette (59). In the first step, the Janus cassette was amplified from genomic DNA, obtained using a MasterPure DNA purification kit (Epicentre/Lucigen), from strain P2408 (60), with flanking regions (∼1 kb) upstream and downstream of the *dltB* gene added via isothermal assembly. Strain P2406 was then transformed with the PCR product, and the transformants were selected on TS agar-kanamycin (125 μg/ml) plates. A PCR-confirmed mutant, P2552 (*dltB*::Janus Kan^r^ Strep^s^), was selected to make an in-frame deletion. The mutant was transformed with a PCR product using primers *F1-dltB*/*R7-dltB* and *F8-dltB*/*R6-dltB* and genomic DNA obtained from P2406 and selected on TS agar-streptomycin (200 μg/ml) to generate strain P2555 (Δ*dltB* Strep^r^ Kan^s^). The in-frame knockout strain (the Δ*dltB* strain) has a scar containing the first and the last 5-amino-acid coding sequences of the *dltB* gene.

The corrected mutant, P2556 (the *dltB^+^* mutant), was constructed by transforming strain P2552 with a PCR product generated using primers *F1-dltB* and *R6-dltB* to amplify genomic DNA from strain P2406, and the transformants were selected on TS agar-streptomycin (200 μg/ml). The *dlt* locus knockout mutant was constructed as described above, with the primers used being listed in Table S2 in the supplemental material. Neither the *dltB*- nor *dlt*-negative mutant constructs showed a growth defect in nutrient medium.

10.1128/mBio.01032-19.2 TABLE S2 Primers. Download Table S2, DOCX file, 0.01 MB. Copyright © 2019 Zafar et al. 2019 Zafar et al. This content is distributed under the terms of the Creative Commons Attribution 4.0 International license.

The *ciaRH* knockout strain P2597 (*ciaRH*::*erm*) was constructed by transforming genomic DNA obtained from an isolate with a *ciaRH*::*erm* cassette into P2406 (15). The resulting transformants were selected on TS agar plates supplemented with erythromycin (2 μg/ml) and 5% blood. A second back-transformation was carried out in P2406 with genomic DNA obtained from one of the transformants, and isolates were selected on plates as described above to generate strain P2597 (*ciaRH*::*erm* Erm^r^ Strep^r^).

### Shedding and colonization in infant mice.

WT, *tlr2*^−/−^, and *lysM*^−/−^ C57BL/6J mice were obtained from The Jackson Laboratory (Bar Harbor, ME) and bred and maintained in our conventional animal facility at the NYU Langone Medical Center. The pups were housed with their dam (mother) for the duration of the experiment and gained weight like uninfected animals.

At day 4 of life, the pups were given an intranasal (i.n.) inoculation, without anesthesia, containing ∼2,000 CFU of S. pneumoniae suspended in 3 μl of PBS, as described previously (15, 16). Shedding was quantified by gently tapping the nares (20 taps/pup) on a TS agar plate supplemented with streptomycin (200 μg/ml) or kanamycin (125 μg/ml) to prevent the growth of contaminants, followed by spreading of the secretions over the agar surface with a sterile cotton-tipped swab. Because of the day-to-day variability in the numbers of shed bacteria, values were obtained daily for 5 days and pooled. To control for environment effects, shedding of different strains was compared for pups within the same litter. To measure colonization density, we euthanized the pups at the ages indicated below by CO_2_ asphyxiation, followed by cardiac puncture. The upper respiratory tract (URT) was lavaged with 200 μl of sterile PBS from a needle inserted into the trachea, and fluid was collected from the nares. The limit of detection in lavage fluids was 33 CFU/ml.

### Transmission in infant mice.

The pneumococcal monoinfection transmission model was described in previous studies (15). Briefly, half of the pups in a litter were randomly selected and, at day 4 of life, infected with the S. pneumoniae strain indicated above. These index mice were then returned to the dam and the other uninfected pups (contact mice) and cohoused for 10 days postinfection. To detect bacterial transmission from the index pups to the contact pups, all pups were euthanized at the age of 14 days, and nasal lavage fluid specimens were cultured.

### Transposon library preparation and *in vivo* selection.

Library construction using the mariner transposon was carried out as previously described (20) with slight modifications. Briefly, 28 independent libraries were generated, with each library containing ∼500 mutants with transposon insertion mutations in nonessential genes. On a given day, a transposon pool was thawed and grown at 37°C, and two 4-day-old pups (biological replicates) were inoculated i.n. with 4,000 to 8,000 CFU of transposon mutants, such that each pup received an ∼10-fold excess of the random mutants (input). Pilot experiments with various inocula confirmed an infectious dose of <10 CFU and the lack of a population bottleneck (the systematic loss of clones) when pups were colonized as described above (data not shown).

After infection, pneumococcal shedding over 5 days was carried out as described above. After shed pneumococci were quantified, colonies were removed with a swab, transferred to 2 ml of TS broth, and incubated at 37°C for an hour, before adding glycerol at a final concentration 20% for storage at −80°C until further processing. Shedding for the two biological replicates was kept separate and was carried out over 5 days. Afterwards, shed bacteria collected over all 5 days (≥3,000 total CFU) were pooled and pelleted by centrifugation at 4,300 × g for 10 min. Genomic DNA was isolated as described above, and samples were sequenced as described previously (20) (output I). A cleanup step was added to the ligation mixture to remove excess adaptor using AMPure XP beads (Beckman Coulter), before using PCR to amplify the transposon insertion site.

At day 9 of life, pups infected with S. pneumoniae transposon library mutants were sacrificed and nasal lavage specimens were collected in a 200-μl volume as described above. Ten microliters was used to determine the colonization density, and the remainder of the lavage fluid was plated on TS agar-streptomycin (200 μg/ml) supplemented with catalase and incubated overnight at 37°C. On the next day, the colonies were collected by swabbing and transferred to 5 ml sterile PBS (output II). Genomic DNA was isolated, and samples were prepared as described above.

### Sequencing and bioinformatics analysis.

Transposon junction DNA fragments were subjected to single-end 50-rapid-run sequencing on an Illumina HiSeq 2500 instrument (Illumina, San Diego, CA). The resulting reads were filtered, mapped, and normalized as described previously (61). Briefly, raw sequencing reads were searched for the transposon sequence ACAGGTTG, and the reads without this sequence were discarded. The FASTX-Toolkit (http://hannonlab.cshl.edu/fastx_toolkit/) was used to debarcode the reads based on the barcode sequences at the 5′ ends, and the reads that became shorter than 12 bp after barcode trimming were discarded. The debarcoded reads were then processed using an IN-Seq pipeline as published previously (61). Specifically, reads were aligned to the S. pneumoniae TIGR4 reference genome using the Bowtie program (62), and insertion sites were called for each sample based on the alignment counts. Only uniquely mapped reads with no more than one mismatch were used. Insertion sites within 80% of the gene length from the transcription start sites were treated as candidates that could affect gene functions. Finally, TnseqDiff (23) was used to compare the insertions between output and input samples to identify genes potentially essential under different conditions. Comparison was carried out for the following groups: input versus shedding and input versus nasal lavage.

### Antimicrobial sensitivity assay.

Stocks of nisin (catalog number M5764-1G; Sigma) were prepared in 0.05% acetic acid (10 mg/ml), stocks of bacitracin (catalog number B5150-250KU; Sigma) were prepared in 1 M HCl (50 mg/ml), and stocks of hen egg white lysozyme (Roche) were prepared in distilled water (20 mg/ml). To determine susceptibility to these antimicrobial peptides, strains were grown in TS broth to mid-log phase and diluted 200-fold in a microtiter plate containing a 2-fold concentration gradient of either nisin or bacitracin, with the final volume being 200 μl. The plate was incubated at 37°C in 5% CO_2_ for 3 h, after which serial dilutions were plated on TS agar plates and incubated at 37°C in 5% CO_2_ overnight. Percent survival was calculated by counting the number of CFU at various concentrations of the antimicrobial peptide versus that for the vehicle control. Each experiment was repeated at least 4 times.

To test sensitivity to lysozyme, strains were grown as mentioned above and diluted 200-fold in a microtiter plate containing either 500 or 1,000 μg/ml lysozyme, with the final volume being 200 μl. Incubation, serial dilutions, and calculation of percent survival were as described above.

### Flow cytometry.

Neutrophils present in the nasopharynx were quantified as previously described (18). Briefly, nasal lavage samples from individual pups were pelleted by centrifugation at 500 × *g* for 5 min. Samples were resuspended in 50 μl ACK lysis buffer (Thermo Fisher Scientific) and incubated for 5 min at room temperature. Afterwards, 200 μl of PBS was added to the samples, and cells were pelleted as described above and resuspended in PBS containing 1% bovine serum albumin (BSA). Samples were stained with a LIVE/DEAD Fixable Aqua dead cell stain kit (Invitrogen, Thermo Fisher Scientific). Next, samples were blocked with a 1:200 dilution of a rat anti-mouse CD16/32 (clone 93; BioLegend). Cells were stained for 30 min at 4°C with fluorophore-conjugated antibodies (diluted 1:150) against the following surface markers: CD11b-V450 (BD), Ly6G-peridinin chlorophyll protein (PerCP)-Cy5.5 (BD), and CD45-allophycocyanin (APC)-Cy7 (BD). Samples were run on a BD LSR II flow cytometer and analyzed with BD FACSDiva software.

### qRT-PCR.

The upper respiratory tract of the pups was lavaged using RLT lysis buffer (Qiagen) to obtain RNA from the epithelium. Total RNA and cDNA generation was carried out as previously described (60). Quantitative reverse transcription-PCRs (qRT-PCRs) were performed using Power SYBR green master mix (Applied Biosystems), ∼10 ng cDNA, and 0.5 mM primers per reaction mixture. Samples were run in duplicate, and each experiment run was repeated. Samples were run on a CFX384 Touch real-time PCR detection system (Bio-Rad). Primers directed toward the GAPDH (glyceraldehyde-3-phosphate dehydrogenase) gene were used as an internal control. RNA expression was quantified using the ΔΔ*C_T_* threshold cycle (*C_T_*) method. The primers used in this study were previously described (60, 63).

To determine the *dltA* transcript level upon treatment with lysozyme, the bacterial strains were grown in TS broth at 37°C until they reached mid-log phase (OD_620_ = 0.5), back-diluted 4-fold in fresh TS broth containing either 1 mg/ml lysozyme or water (mock treatment), and grown statically again at 37°C for 1 h. Afterwards, the cultures were spun down at 4°C at 4,300 × *g* for 10 min. Samples were treated with RNAprotect Bacteria Reagent (Qiagen), and RNA was isolated using an AllPrep bacterial DNA/RNA/protein kit (Qiagen) following the manufacturer’s protocol. cDNA and qRT-PCR were carried out as described above using a primer set against *dltA* and a primer set against DNA gyrase (*gyr*) as an internal control.

### Statistical analysis.

All statistical analyses were performed using GraphPad Prism (version 7.0) software (GraphPad Software, Inc., San Diego, CA). Unless otherwise specified, differences were determined using the Mann-Whitney U test (comparing two groups) or the Kruskal-Wallis test with Dunn’s postanalysis (comparing multiple groups).

## References

[1] Henriques-Normark B, Tuomanen EI 2013 The pneumococcus: epidemiology, microbiology, and pathogenesis. Cold Spring Harb Perspect Med 3:a010215. doi:10.1101/cshperspect.a010215. 23818515 PMC3685878

[2] Sleeman KL, Daniels L, Gardiner M, Griffiths D, Deeks JJ, Dagan R, Gupta S, Moxon ER, Peto TE, Crook DW 2005 Acquisition of Streptococcus pneumoniae and nonspecific morbidity in infants and their families—a cohort study. Pediatr Infect Dis J 24:121–127. doi:10.1097/01.inf.0000151030.10159.b1. 15702039

[3] Givon-Lavi N, Fraser D, Porat N, Dagan R 2002 Spread of Streptococcus pneumoniae and antibiotic-resistant S. pneumoniae from day-care center attendees to their younger siblings. J Infect Dis 186:1608–1614. doi:10.1086/345556. 12447737

[4] Weinberger DM, Dagan R, Givon-Lavi N, Regev-Yochay G, Malley R, Lipsitch M 2008 Epidemiologic evidence for serotype-specific acquired immunity to pneumococcal carriage. J Infect Dis 197:1511–1518. doi:10.1086/587941. 18471062

[5] Leggett HC, Cornwallis CK, Buckling A, West SA 2017 Growth rate, transmission mode and virulence in human pathogens. Philos Trans R Soc Lond B Biol Sci 372:20160094. doi:10.1098/rstb.2016.0094. 28289261 PMC5352820

[6] Herfst S, Bohringer M, Karo B, Lawrence P, Lewis NS, Mina MJ, Russell CJ, Steel J, de Swart RL, Menge C 2017 Drivers of airborne human-to-human pathogen transmission. Curr Opin Virol 22:22–29. doi:10.1016/j.coviro.2016.11.006. 27918958 PMC7102691

[7] Weiser JN, Ferreira DM, Paton JC 2018 Streptococcus pneumoniae: transmission, colonization and invasion. Nat Rev Microbiol 16:355–367. doi:10.1038/s41579-018-0001-8. 29599457 PMC5949087

[8] Bogaert D, De Groot R, Hermans PW 2004 *Streptococcus pneumoniae* colonisation: the key to pneumococcal disease. Lancet Infect Dis 4:144–154. doi:10.1016/S1473-3099(04)00938-7. 14998500

[9] Simell B, Auranen K, Käyhty H, Goldblatt D, Dagan R, O’Brien KL, Pneumococcal Carriage Group. 2012 The fundamental link between pneumococcal carriage and disease. Expert Rev Vaccines 11:841–855. doi:10.1586/erv.12.53. 22913260

[10] O'Brien KL, Wolfson LJ, Watt JP, Henkle E, Deloria-Knoll M, McCall N, Lee E, Mulholland K, Levine OS, Cherian T, Dis H 2009 Burden of disease caused by Streptococcus pneumoniae in children younger than 5 years: global estimates. Lancet 374:893–902. doi:10.1016/S0140-6736(09)61204-6. 19748398

[11] McCullers JA 2006 Insights into the interaction between influenza virus and pneumococcus. Clin Microbiol Rev 19:571–582. doi:10.1128/CMR.00058-05. 16847087 PMC1539103

[12] CDC. 2015 Pneumococcal disease. Epidemiology and prevention of vaccine-preventable diseases. CDC, Atlanta, GA https://www.cdc.gov/vaccines/pubs/pinkbook/pneumo.html.

[13] van der Poll T, Opal SM 2009 Pathogenesis, treatment, and prevention of pneumococcal pneumonia. Lancet 374:1543–1556. doi:10.1016/S0140-6736(09)61114-4. 19880020

[14] WHO. 2017 WHO publishes list of bacteria for which new antibiotics are urgently needed. WHO, Geneva, Switzerland.

[15] Zafar MA, Kono M, Wang Y, Zangari T, Weiser JN 2016 Infant mouse model for the study of shedding and transmission during Streptococcus pneumoniae monoinfection. Infect Immun 84:2714–2722. doi:10.1128/IAI.00416-16. 27400721 PMC4995895

[16] Richard AL, Siegel SJ, Erikson J, Weiser JN 2014 TLR2 signaling decreases transmission of *Streptococcus pneumoniae* by limiting bacterial shedding in an infant mouse influenza A co-infection model. PLoS Pathog 10:e1004339. doi:10.1371/journal.ppat.1004339. 25166617 PMC4148449

[17] Kono M, Zafar MA, Zuniga M, Roche AM, Hamaguchi S, Weiser JN 2016 Single cell bottlenecks in the pathogenesis of *Streptococcus pneumoniae*. PLoS Pathog 12:e1005887. doi:10.1371/journal.ppat.1005887. 27732665 PMC5061371

[18] Zafar MA, Wang Y, Hamaguchi S, Weiser JN 2017 Host-to-host transmission of *Streptococcus pneumoniae* is driven by its inflammatory toxin, pneumolysin. Cell Host Microbe 21:73–83. doi:10.1016/j.chom.2016.12.005. 28081446 PMC5267320

[19] Zafar MA, Hamaguchi S, Zangari T, Cammer M, Weiser JN 2017 Capsule type and amount affect shedding and transmission of Streptococcus pneumoniae. mBio 8:e00989-17. doi:10.1128/mBio.00989-17. 28830943 PMC5565965

[20] van Opijnen T, Lazinski DW, Camilli A 2017 Genome-wide fitness and genetic interactions determined by Tn-seq, a high-throughput massively parallel sequencing method for microorganisms. Curr Protoc Microbiol 36:1e.3.1–1e.3.24. doi:10.1002/9780471729259.mc01e03s36. PMC4696536 25641100

[21] Rajagopal M, Walker S 2017 Envelope structures of Gram-positive bacteria. Curr Top Microbiol Immunol 404:1–44. doi:10.1007/82_2015_5021. 26919863 PMC5002265

[22] Percy MG, Grundling A 2014 Lipoteichoic acid synthesis and function in gram-positive bacteria. Annu Rev Microbiol 68:81–100. doi:10.1146/annurev-micro-091213-112949. 24819367

[23] Zhao L, Anderson MT, Wu W, Mobley HLT, Bachman MA 2017 TnseqDiff: identification of conditionally essential genes in transposon sequencing studies. BMC Bioinformatics 18:326. doi:10.1186/s12859-017-1745-2. 28683752 PMC5500955

[24] Wood BM, Santa Maria JP Jr, Matano LM, Vickery CR, Walker S 2018 A partial reconstitution implicates DltD in catalyzing lipoteichoic acid d-alanylation. J Biol Chem 293:17985–17996. doi:10.1074/jbc.RA118.004561. 30237166 PMC6240853

[25] Perego M, Glaser P, Minutello A, Strauch MA, Leopold K, Fischer W 1995 Incorporation of d-alanine into lipoteichoic acid and wall teichoic acid in Bacillus subtilis. J Biol Chem 270:15598–15606. doi:10.1074/jbc.270.26.15598. 7797557

[26] Ma D, Wang Z, Merrikh CN, Lang KS, Lu P, Li X, Merrikh H, Rao Z, Xu W 2018 Crystal structure of a membrane-bound O-acyltransferase. Nature 562:286–290. doi:10.1038/s41586-018-0568-2. 30283133 PMC6529733

[27] Kovács M, Halfmann A, Fedtke I, Heintz M, Peschel A, Vollmer W, Hakenbeck R, Brückner R 2006 A functional dlt operon, encoding proteins required for incorporation of d-alanine in teichoic acids in Gram-positive bacteria, confers resistance to cationic antimicrobial peptides in *Streptococcus pneumoniae*. J Bacteriol 188:5797–5805. doi:10.1128/JB.00336-06. 16885447 PMC1540085

[28] Storm DR 1974 Mechanism of bacitracin action: a specific lipid-peptide interaction. Ann N Y Acad Sci 235:387–398. doi:10.1111/j.1749-6632.1974.tb43278.x. 4368896

[29] Seo HS, Michalek SA, Nahm MH 2008 Lipoteichoic acid is important in innate immune responses to Gram-positive bacteria. Infect Immun 76:206–213. doi:10.1128/IAI.01140-07. 17954723 PMC2223632

[30] Han SH, Kim JH, Martin M, Michalek SM, Nahm MH 2003 Pneumococcal lipoteichoic acid (LTA) is not as potent as staphylococcal LTA in stimulating Toll-like receptor 2. Infect Immun 71:5541–5548. doi:10.1128/iai.71.10.5541-5548.2003. 14500472 PMC201083

[31] Travassos LH, Girardin SE, Philpott DJ, Blanot D, Nahori MA, Werts C, Boneca IG 2004 Toll-like receptor 2-dependent bacterial sensing does not occur via peptidoglycan recognition. EMBO Rep 5:1000–1006. doi:10.1038/sj.embor.7400248. 15359270 PMC1299148

[32] Draing C, Pfitzenmaier M, Zummo S, Mancuso G, Geyer A, Hartung T, von Aulock S 2006 Comparison of lipoteichoic acid from different serotypes of Streptococcus pneumoniae. J Biol Chem 281:33849–33859. doi:10.1074/jbc.M602676200. 16943191

[33] Cole A, Liao H, Stuchlik O, Tilan J, Pohl J, Ganz T 2002 Cationic polypeptides are required for antibacterial activity of human airway fluid. J Immunol 169:6985–6991. doi:10.4049/jimmunol.169.12.6985. 12471133

[34] Davis K, Akinbi H, Standish A, Weiser J 2008 Resistance to mucosal lysozyme compensates for the fitness deficit of peptidoglycan modifications by *Streptococcus pneumoniae*. PLoS Pathog 4:e1000241. doi:10.1371/journal.ppat.1000241. 19079576 PMC2587705

[35] Guariglia-Oropeza V, Helmann JD 2011 Bacillus subtilis sigma(V) confers lysozyme resistance by activation of two cell wall modification pathways, peptidoglycan O-acetylation and d-alanylation of teichoic acids. J Bacteriol 193:6223–6232. doi:10.1128/JB.06023-11. 21926231 PMC3209214

[36] Woods EC, Nawrocki KL, Suarez JM, McBride SM 2016 The Clostridium difficile Dlt pathway is controlled by the extracytoplasmic function sigma factor sigmaV in response to lysozyme. Infect Immun 84:1902–1916. doi:10.1128/IAI.00207-16. 27068095 PMC4907151

[37] Le Jeune A, Torelli R, Sanguinetti M, Giard JC, Hartke A, Auffray Y, Benachour A 2010 The extracytoplasmic function sigma factor SigV plays a key role in the original model of lysozyme resistance and virulence of Enterococcus faecalis. PLoS One 5:e9658. doi:10.1371/journal.pone.0009658. 20300180 PMC2836378

[38] Mazda Y, Kawada-Matsuo M, Kanbara K, Oogai Y, Shibata Y, Yamashita Y, Miyawaki S, Komatsuzawa H 2012 Association of CiaRH with resistance of Streptococcus mutans to antimicrobial peptides in biofilms. Mol Oral Microbiol 27:124–135. doi:10.1111/j.2041-1014.2012.00637.x. 22394470

[39] Griffin MR, Zhu Y, Moore MR, Whitney CG, Grijalva CG 2013 U.S. hospitalizations for pneumonia after a decade of pneumococcal vaccination. N Engl J Med 369:155–163. doi:10.1056/NEJMoa1209165. 23841730 PMC4877190

[40] Whitney C, Farley M, Hadler J, Harrison L, Bennett N, Lynfield R, Reingold A, Cieslak P, Pilishvili T, Jackson D, Facklam R, Jorgensen J, Schuchat A, Active Bacterial Core Surveillance of the Emerging Infections Program Network. 2003 Decline in invasive pneumococcal disease after the introduction of protein-polysaccharide conjugate vaccine. N Engl J Med 348:1737–1746. doi:10.1056/NEJMoa022823. 12724479

[41] Lexau C, Lynfield R, Danila R, Pilishvili T, Facklam R, Farley M, Harrison L, Schaffner W, Reingold A, Bennett N, Hadler J, Cieslak P, Whitney C, Active Bacterial Core Surveillance Team. 2005 Changing epidemiology of invasive pneumococcal disease among older adults in the era of pediatric pneumococcal conjugate vaccine. JAMA 294:2043–2051. doi:10.1001/jama.294.16.2043. 16249418

[42] Pilishvili T, Lexau C, Farley MM, Hadler J, Harrison LH, Bennett NM, Reingold A, Thomas A, Schaffner W, Craig AS, Smith PJ, Beall BW, Whitney CG, Moore MR, Active Bacterial Core Surveillance/Emerging Infections Program Network. 2010 Sustained reductions in invasive pneumococcal disease in the era of conjugate vaccine. J Infect Dis 201:32–41. doi:10.1086/648593. 19947881

[43] Black S, Shinefield H, Fireman B, Lewis E, Ray P, Hansen J, Elvin L, Ensor K, Hackell J, Siber G, Malinoski F, Madore D, Chang I, Kohberger R, Watson W, Austrian R, Edwards K 2000 Efficacy, safety and immunogenicity of heptavalent pneumococcal conjugate vaccine in children. Pediatr Infect Dis J 19:187–195. doi:10.1097/00006454-200003000-00003. 10749457

[44] Hava DL, Camilli A 2002 Large-scale identification of serotype 4 *Streptococcus pneumoniae* virulence factors. Mol Microbiol 45:1389–1406. 12207705 PMC2788772

[45] Molzen TE, Burghout P, Bootsma HJ, Brandt CT, van der Gaast-de Jongh CE, Eleveld MJ, Verbeek MM, Frimodt-Møller N, Østergaard C, Hermans PWM 2011 Genome-wide identification of Streptococcus pneumoniae genes essential for bacterial replication during experimental meningitis. Infect Immun 79:288–297. doi:10.1128/IAI.00631-10. 21041497 PMC3019918

[46] Carter R, Wolf J, van Opijnen T, Muller M, Obert C, Burnham C, Mann B, Li Y, Hayden RT, Pestina T, Persons D, Camilli A, Flynn PM, Tuomanen EI, Rosch JW 2014 Genomic analyses of pneumococci from children with sickle cell disease expose host-specific bacterial adaptations and deficits in current interventions. Cell Host Microbe 15:587–599. doi:10.1016/j.chom.2014.04.005. 24832453 PMC4066559

[47] Verhagen LM, de Jonge MI, Burghout P, Schraa K, Spagnuolo L, Mennens S, Eleveld MJ, van der Gaast-de Jongh CE, Zomer A, Hermans PW, Bootsma HJ 2014 Genome-wide identification of genes essential for the survival of *Streptococcus pneumoniae* in human saliva. PLoS One 9:e89541. doi:10.1371/journal.pone.0089541. 24586856 PMC3934895

[48] Hausdorff WP, Bryant J, Paradiso PR, Siber GR 2000 Which pneumococcal serogroups cause the most invasive disease: implications for conjugate vaccine formulation and use, part I. Clin Infect Dis 30:100–121. doi:10.1086/313608. 10619740

[49] Babl FE, Pelton SI, Theodore S, Klein JO 2001 Constancy of distribution of serogroups of invasive pneumococcal isolates among children: experience during 4 decades. Clin Infect Dis 32:1155–1161. doi:10.1086/319750. 11283804

[50] Hausdorff WP, Feikin DR, Klugman KP 2005 Epidemiological differences among pneumococcal serotypes. Lancet Infect Dis 5:83–93. doi:10.1016/S1473-3099(05)70083-9. 15680778

[51] Kang JY, Nan X, Jin MS, Youn SJ, Ryu YH, Mah S, Han SH, Lee H, Paik SG, Lee JO 2009 Recognition of lipopeptide patterns by Toll-like receptor 2-Toll-like receptor 6 heterodimer. Immunity 31:873–884. doi:10.1016/j.immuni.2009.09.018. 19931471

[52] Gisch N, Kohler T, Ulmer AJ, Muthing J, Pribyl T, Fischer K, Lindner B, Hammerschmidt S, Zahringer U 2013 Structural reevaluation of Streptococcus pneumoniae lipoteichoic acid and new insights into its immunostimulatory potency. J Biol Chem 288:15654–15667. doi:10.1074/jbc.M112.446963. 23603911 PMC3668725

[53] Hashimoto M, Tawaratsumida K, Kariya H, Kiyohara A, Suda Y, Krikae F, Kirikae T, Gotz F 2006 Not lipoteichoic acid but lipoproteins appear to be the dominant immunobiologically active compounds in Staphylococcus aureus. J Immunol 177:3162–3169. doi:10.4049/jimmunol.177.5.3162. 16920954

[54] Vollmer W, Tomasz A 2000 The pgdA gene encodes for a peptidoglycan N-acetylglucosamine deacetylase in Streptococcus pneumoniae. J Biol Chem 275:20496–20501. doi:10.1074/jbc.M910189199. 10781617

[55] Vollmer W, Tomasz A 2002 Peptidoglycan *N*-acetylglucosamine deacetylase, a putative virulence factor in *Streptococcus pneumoniae*. Infect Immun 70:176–178. doi:10.1128/IAI.70.12.7176-7178.2002. PMC133073 12438406

[56] Crisostomo M, Vollmer W, Kharat A, Inhulsen S, Gehre F, Buckenmaier S, Tomasz A 2006 Attenuation of penicillin resistance in a peptidoglycan O-acetyl transferase mutant of *Streptococcus pneumoniae*. Mol Microbiol 61:1497–1509. doi:10.1111/j.1365-2958.2006.05340.x. 16968223

[57] Saar-Dover R, Bitler A, Nezer R, Shmuel-Galia L, Firon A, Shimoni E, Trieu-Cuot P, Shai Y 2012 d-Alanylation of lipoteichoic acids confers resistance to cationic peptides in group B streptococcus by increasing the cell wall density. PLoS Pathog 8:e1002891. doi:10.1371/journal.ppat.1002891. 22969424 PMC3435245

[58] National Institutes of Health. 2015 Public Health Service policy on humane care and use of laboratory animals. National Institutes of Health, Bethesda, MD.

[59] Sung C, Li H, Claverys J, Morrison D 2001 An rpsL cassette, Janus, for gene replacement through negative selection in *Streptococcus pneumoniae*. Appl Environ Microbiol 67:5190–5196. doi:10.1128/AEM.67.11.5190-5196.2001. 11679344 PMC93289

[60] Lemon JK, Weiser JN 2015 Degradation products of the extracellular pathogen *Streptococcus pneumoniae* access the cytosol via its pore-forming toxin. mBio 6:e02110-14. doi:10.1128/mBio.02110-14. 25604786 PMC4313911

[61] Goodman AL, Wu M, Gordon JI 2011 Identifying microbial fitness determinants by insertion sequencing using genome-wide transposon mutant libraries. Nat Protoc 6:1969–1980. doi:10.1038/nprot.2011.417. 22094732 PMC3310428

[62] Langmead B, Trapnell C, Pop M, Salzberg SL 2009 Ultrafast and memory-efficient alignment of short DNA sequences to the human genome. Genome Biol 10:R25. doi:10.1186/gb-2009-10-3-r25. 19261174 PMC2690996

[63] Siegel SJ, Tamashiro E, Weiser JN 2015 Clearance of pneumococcal colonization in infants is delayed through altered macrophage trafficking. PLoS Pathog 11:e1005004. doi:10.1371/journal.ppat.1005004. 26107875 PMC4479461

[64] Tettelin H, Nelson KE, Paulsen IT, Eisen JA, Read TD, Peterson S, Heidelberg J, DeBoy RT, Haft DH, Dodson RJ, Durkin AS, Gwinn M, Kolonay JF, Nelson WC, Peterson JD, Umayam LA, White O, Salzberg SL, Lewis MR, Radune D, Holtzapple E, Khouri H, Wolf AM, Utterback TR, Hansen CL, McDonald LA, Feldblyum TV, Angiuoli S, Dickinson T, Hickey EK, Holt IE, Loftus BJ, Yang F, Smith HO, Venter JC, Dougherty BA, Morrison DA, Hollingshead SK, Fraser CM 2001 Complete genome sequence of a virulent isolate of Streptococcus pneumoniae. Science 293:498–506. doi:10.1126/science.1061217. 11463916

[65] Basavanna S, Khandavilli S, Yuste J, Cohen JM, Hosie AH, Webb AJ, Thomas GH, Brown JS 2009 Screening of Streptococcus pneumoniae ABC transporter mutants demonstrates that LivJHMGF, a branched-chain amino acid ABC transporter, is necessary for disease pathogenesis. Infect Immun 77:3412–3423. doi:10.1128/IAI.01543-08. 19470745 PMC2715661

[66] Berry AM, Lock RA, Thomas SM, Rajan DP, Hansman D, Paton JC 1994 Cloning and nucleotide sequence of the Streptococcus pneumoniae hyaluronidase gene and purification of the enzyme from recombinant Escherichia coli. Infect Immun 62:1101–1108. 8112843 10.1128/iai.62.3.1101-1108.1994 PMC186229

[67] Li S, Kelly SJ, Lamani E, Ferraroni M, Jedrzejas MJ 2000 Structural basis of hyaluronan degradation by Streptococcus pneumoniae hyaluronate lyase. EMBO J 19:1228–1240. doi:10.1093/emboj/19.6.1228. 10716923 PMC305664

[68] Yother J 2011 Capsules of Streptococcus pneumoniae and other bacteria: paradigms for polysaccharide biosynthesis and regulation. Annu Rev Microbiol 65:563–581. doi:10.1146/annurev.micro.62.081307.162944. 21721938

[69] Marion C, Limoli D, Bobulsky G, Abraham J, Burnaugh A, King S 2009 Identification of a pneumococcal glycosidase that modifies O-linked glycans. Infect Immun 77:1389–1396. doi:10.1128/IAI.01215-08. 19139197 PMC2663135

[70] Molina R, González A, Stelter M, Pérez-Dorado I, Kahn R, Morales M, Moscoso M, Campuzano S, Campillo NE, Mobashery S, García JL, García P, Hermoso JA 2009 Crystal structure of CbpF, a bifunctional choline-binding protein and autolysis regulator from Streptococcus pneumoniae. EMBO Rep 10:246–251. doi:10.1038/embor.2008.245. 19165143 PMC2658566

[71] Hava D, Hemsley C, Camilli A 2003 Transcriptional regulation in the *Streptococcus pneumoniae rlrA* pathogenicity islet by RlrA. J Bacteriol 185:413–421. doi:10.1128/jb.185.2.413-421.2003. 12511486 PMC145342

[72] Barocchi M, Ries J, Zogaj X, Hemsley C, Albiger B, Kanth A, Dahlberg S, Fernebro J, Moschioni M, Masignani V, Hultenby K, Taddei A, Beiter K, Wartha F, von Euler A, Covacci A, Holden D, Normark S, Rappuoli R, Henriques-Normark B 2006 A pneumococcal pilus influences virulence and host inflammatory responses. Proc Natl Acad Sci U S A 103:2857–2862. doi:10.1073/pnas.0511017103. 16481624 PMC1368962

[73] Kahya HF, Andrew PW, Yesilkaya H 2017 Deacetylation of sialic acid by esterases potentiates pneumococcal neuraminidase activity for mucin utilization, colonization and virulence. PLoS Pathog 13:e1006263. doi:10.1371/journal.ppat.1006263. 28257499 PMC5352144

[74] Gutiérrez-Fernández J, Saleh M, Alcorlo M, Gómez-Mejía A, Pantoja-Uceda D, Treviño MA, Voß F, Abdullah MR, Galán-Bartual S, Seinen J, Sánchez-Murcia PA, Gago F, Bruix M, Hammerschmidt S, Hermoso JA 2016 Modular architecture and unique teichoic acid recognition features of choline-binding protein L (CbpL) contributing to pneumococcal pathogenesis. Sci Rep 6:38094. doi:10.1038/srep38094. 27917891 PMC5137146

[75] Hermans PW, Adrian PV, Albert C, Estevao S, Hoogenboezem T, Luijendijk IH, Kamphausen T, Hammerschmidt S 2006 The streptococcal lipoprotein rotamase A (SlrA) is a functional peptidyl-prolyl isomerase involved in pneumococcal colonization. J Biol Chem 281:968–976. doi:10.1074/jbc.M510014200. 16260779

[76] Pribyl T, Moche M, Dreisbach A, Bijlsma JJ, Saleh M, Abdullah MR, Hecker M, van Dijl JM, Becher D, Hammerschmidt S 2014 Influence of impaired lipoprotein biogenesis on surface and exoproteome of Streptococcus pneumoniae. J Proteome Res 13:650–667. doi:10.1021/pr400768v. 24387739

[77] De Las Rivas B, García JL, López R, García P 2002 Purification and polar localization of pneumococcal LytB, a putative endo-beta-N-acetylglucosaminidase: the chain-dispersing murein hydrolase. J Bacteriol 184:4988–5000. doi:10.1128/jb.184.18.4988-5000.2002. 12193614 PMC135310

[78] Saleh M, Bartual SG, Abdullah MR, Jensch I, Asmat TM, Petruschka L, Pribyl T, Gellert M, Lillig CH, Antelmann H, Hermoso JA, Hammerschmidt S 2013 Molecular architecture of Streptococcus pneumoniae surface thioredoxin-fold lipoproteins crucial for extracellular oxidative stress resistance and maintenance of virulence. EMBO Mol Med 5:1852–1870. doi:10.1002/emmm.201202435. 24136784 PMC3914529

[79] Khan MN, Pichichero ME 2012 Vaccine candidates PhtD and PhtE of Streptococcus pneumoniae are adhesins that elicit functional antibodies in humans. Vaccine 30:2900–2907. doi:10.1016/j.vaccine.2012.02.023. 22349524 PMC3490617

[80] Fasching CE, Grossman T, Corthesy B, Plaut AG, Weiser JN, Janoff EN 2007 Impact of the molecular form of immunoglobulin A on functional activity in defense against Streptococcus pneumoniae. Infect Immun 75:1801–1810. doi:10.1128/IAI.01758-06. 17261616 PMC1865688

[81] Janoff EN, Rubins JB, Fasching C, Charboneau D, Rahkola JT, Plaut AG, Weiser JN 2014 Pneumococcal IgA1 protease subverts specific protection by human IgA1. Mucosal Immunol 7:249–256. doi:10.1038/mi.2013.41. 23820749 PMC4456019

[82] Du Y, He YX, Zhang ZY, Yang YH, Shi WW, Frolet C, Di Guilmi AM, Vernet T, Zhou CZ, Chen Y 2011 Crystal structure of the mucin-binding domain of Spr1345 from Streptococcus pneumoniae. J Struct Biol 174:252–257. doi:10.1016/j.jsb.2010.10.016. 21055474

[83] Bumbaca D, Littlejohn JE, Nayakanti H, Lucas AH, Rigden DJ, Galperin MY, Jedrzejas MJ 2007 Genome-based identification and characterization of a putative mucin-binding protein from the surface of Streptococcus pneumoniae. Proteins 66:547–558. doi:10.1002/prot.21205. 17115425

[84] Garcia P, Paz Gonzalez M, Garcia E, Garcia JL, Lopez R 1999 The molecular characterization of the first autolytic lysozyme of Streptococcus pneumoniae reveals evolutionary mobile domains. Mol Microbiol 33:128–138. doi:10.1046/j.1365-2958.1999.01455.x. 10411730

[85] Iyer R, Camilli A 2007 Sucrose metabolism contributes to in vivo fitness of Streptococcus pneumoniae. Mol Microbiol 66:1–13. doi:10.1111/j.1365-2958.2007.05878.x. 17880421 PMC2790422

[86] Marion C, Stewart JM, Tazi MF, Burnaugh AM, Linke CM, Woodiga SA, King SJ 2012 Streptococcus pneumoniae can utilize multiple sources of hyaluronic acid for growth. Infect Immun 80:1390–1398. doi:10.1128/IAI.05756-11. 22311922 PMC3318431

[87] Culurgioni S, Tang M, Walsh MA 2017 Structural characterization of the Streptococcus pneumoniae carbohydrate substrate-binding protein SP0092. Acta Crystallogr F Struct Biol Commun 73:54–61. doi:10.1107/S2053230X16020252. 28045395 PMC5287374

[88] Brown JS, Ogunniyi AD, Woodrow MC, Holden DW, Paton JC 2001 Immunization with components of two iron uptake ABC transporters protects mice against systemic Streptococcus pneumoniae infection. Infect Immun 69:6702–6706. doi:10.1128/IAI.69.11.6702-6706.2001. 11598041 PMC100046

[89] Brown JS, Gilliland SM, Holden DW 2001 A *Streptococcus pneumoniae* pathogenicity island encoding an ABC transporter involved in iron uptake and virulence. Mol Microbiol 40:572–585. doi:10.1046/j.1365-2958.2001.02414.x. 11359564

[90] Beiter K, Wartha F, Albiger B, Normark S, Zychlinsky A, Henriques-Normark B 2006 An endonuclease allows *Streptococcus pneumoniae* to escape from neutrophil extracellular traps. Curr Biol 16:401–407. doi:10.1016/j.cub.2006.01.056. 16488875

[91] Cox GB, Rosenberg H, Downie JA, Silver S 1981 Genetic analysis of mutants affected in the Pst inorganic phosphate transport system. J Bacteriol 148:1–9. 7026529 10.1128/jb.148.1.1-9.1981 PMC216160

[92] Soualhine H, Brochu V, Menard F, Papadopoulou B, Weiss K, Bergeron MG, Legare D, Drummelsmith J, Ouellette M 2005 A proteomic analysis of penicillin resistance in Streptococcus pneumoniae reveals a novel role for PstS, a subunit of the phosphate ABC transporter. Mol Microbiol 58:1430–1440. doi:10.1111/j.1365-2958.2005.04914.x. 16313627

[93] Di Guilmi AM, Dessen A, Dideberg O, Vernet T 2003 The glycosyltransferase domain of penicillin-binding protein 2a from Streptococcus pneumoniae catalyzes the polymerization of murein glycan chains. J Bacteriol 185:4418–4423. doi:10.1128/jb.185.15.4418-4423.2003. 12867450 PMC165775

