# Supplementary material for: Identification of Pneumococcal Factors Affecting Pneumococcal Shedding Shows that the dlt Locus Promotes Inflammation and Transmission
Source: mBio. 2019 Jun 18;10(3):e01032-19. doi: 10.1128/mBio.01032-19 (PMC6581856; doi:10.1128/mBio.01032-19)
Supplement: TABLE S2 [file mBio.01032-19-st002.docx]

| **Primer** | **Sequence 5’🡪3’** |
| --- | --- |
| F1-dltB | TCATGTATTGGGCACCGACC |
| R2-dltB | cattatccattaaaaatcaaacggatGTTGAAAAAACTCCATCATCTCTTA |
| F3-dltB | GAATAAGAGATGATGGAGTTTTTTCAAatccgtttgatttttaatggataatg |
| R4-dltB | CATATTTTATTTCCTTTTATTTTTTAAACCAttatgcttttggacgtttagtacc |
| F5-dltB | cggtactaaacgtccaaaagcataaTGGTTTAAAAAATAAAAGGAAATAAAATATG |
| R6-dltB | AAATACTGCTGGAAGGCTGCT |
| R7-dltB | TTATTTCCTTTTATTTTTTAAACCATTGAAAAAACTCCATCATCTCTTATTC |
| F8-dltB | GAATAAGAGATGATGGAGTTTTTTCAATGGTTTAAAAAATAAAAGGAAATAA |
| F1-dltA-dltD | AAATCGTGTGACGCACCAAG |
| R2-dltA-dltD | attatccattaaaaatcaaacggatTGGTTTATTTGACACAATAGGGATTC |
| F3-dltA-dltD | GAATCCCTATTGTGTCAAATAAACCAatccgtttgatttttaatggataatg |
| R4-dltA-dltD | ATTATATTATCTATTGAAATTCTTTttatgcttttggacgtttagtaccg |
| F5-dltA-dltD | cggtactaaacgtccaaaagcataaAAAGAATTTCAATAGATAATATAATATAG |
| R6-dltA-dltD | TGTTCCCACATGCCAACTGA |
| R7-dltA-dltD | ATTATATTATCTATTGAAATTCTTTTGGTTTATTTGACACAATAGGGATTC |
| F8-dltA-dltD | GAATCCCTATTGTGTCAAATAAACCAAAAGAATTTCAATAGATAATATAATATAG |

Table S2. Primers used in construction of *dlt* mutants*.*
